# The Study of Man in the British Association

**Published:** 1895-10-12

**Authors:** 


### The Study of Man in the British Association.

The annual meeting of the British Association usually brings together some few notable contributions to the study of man, and the meeting at Ipswich last month was no exception to the rule. It is true that Section H received no distinguished foreigners this year; but the communications were, perhaps, on that account the more valuable, as an index of the work which is being done by our own countrymen.

The ethnographic and descriptive papers, though not numerous, were well up to the average. Most important perhaps, though by its very solidity least attractive, was the voluminous fifth report of Dr. Franz Boas on the Indians of British Columbia, whose description he hopes to be able to complete before the Toronto meeting of 1897, with a renewed grant from the association of £100.

A most detailed and very valuable series of photographs of the Andamanese, their customs, arts, and manufactures, was exhibited on behalf of Mr. Maurice Portman, who has made admirable use of the peculiar opportunities which he has had for studying them. Unfortunately no one was able to introduce the subject adequately in his absence.

Messrs. Linklater and Fowler contributed a paper on the Esquimaux of Labrador, with an exhibit of skulls and native manufactures; Mr. A. Montefiore gave an account of the Samoyads of the Tundras or frozen morasses of North Siberia; and Mr. Theodore Bent an ethnological appendix to his geographical report on his journey in Hadramant.

Much interest also was excited by Captain Hinde's account of the cannibal tribes of the Upper Congo, and of the Pigmy race of Central Africa. In the former, the habit of man-eating appears to be inveterate, and of a peculiarly degenerate order; they buy and eat simply for food, and not, as elsewhere, from any motive of religion or magic.

The report of the Ethnographical Survey Committee contained little but preliminary matter, but deserves notice on account of the importance of the work in hand, namely, to collect information from trained observers at a number of selected stations, on the various types and races, which occur in different parts of Great Britain; to determine their origin and distribution; and to collect and codify local peculiarities of custom and tradition. Special anthropometrical reports were presented, in this connection, on East Aberdeenshire, by Mr. T. Gray; and on Suffolk by Dr. Garson; and a study of the Suffolk dialect by Mr. C. G. de Betham.

Anthropometric papers were few. The committees on Physical Deviations of Children and on Anthropometrical Work in Schools presented brief interim reports.

Three other communications of some importance were made in this department. Sir W. H. Flower exhibited four skulls of aborigines of Jamaica, who were exterminated before the middle of the seventeenth century, and whose remains are very rarely found. They seem to resemble the usual type of American native, but the artificial deformation of the head invalidates exacter comparisons.

Dr. Garson described a remarkable but unfortunately much damaged skull, found in the Thames Valley in river gravels which contain paleolithic implements. He contended, on morphological grounds, that the skull was contemporary with the gravels and itself paleolithic. The circumstances of the discovery however, were not very accurately recorded, and the attribution cannot be accepted as entirely certain.

Professor W. M. Flinders Petrie, who presided over the section, gave a full preliminary account of the physical characters of the "New Race" discovered by him in Egypt, of which a notice has already appeared in these pages. The exact affinities of this people, however, still remain to be determined.

Two mornings were devoted to archæological subjects, one to papers on "Stone Implements and the Beginnings of Metal Working," the other to important papers on the well-preserved lake dwellings which have been excavated at Glastonbury, and which probably date from the first or second century B.C.; on the neolithic settlement at Butmir, in Bosnia, which the speaker, Dr. R. Munro, contended also consisted of pile dwellings; by Mr. Arthur Evans on "Primitive European Idols," of which he traced a continuous, related series through South and Central Europe, and even into Russia and Orkney, with important conclusions as to their native, non-Oriental origin, and as to the age and character of the civilisation which they accompany.

Mr. Swainson Cowper's lantern lecture on "The Senams of Tripoli," a numerous series of enormous stone structures like those of Stonehenge, associated with altars and other evidences of religious usage, also contributed material of importance to the early history of the Mediterranean basin.

Folklore, customs, and traditions received a full share of attention in connection with the report of the Ethnographic Survey. The Folklore Society appeared in full force, and Professor Haddon gave an illustrated lecture on "The Survivals of Primitive Beliefs in the West of Ireland and Elsewhere." "Survivals in Language of the Usages of Primitive Warfare" were illustrated by Rev. Hartwell Jones; Mr. Elworthy discussed "The Symbolism of Horns and Crests"; and Mrs. Grove "The Religious and Magical Origin of Certain Dances."

The discussion, which was arranged on "Interference with the Civilisation of Native Races," deserves fuller treatment than can be given here, and will be separately dealt with. The general unanimity of the opinions expressed carried the greater weight, inasmuch as every speaker was required to specify what natives he had himself studied, and all references to individual "civilising" agencies were rigidly excluded.

The opening address also of the President, Professor W. M. Flinders Petrie, dealt with so many and so wide-reaching topics that it cannot be fairly appreciated in a brief abstract.

